# AI chatbots not yet ready for clinical use

**DOI:** 10.3389/fdgth.2023.1161098

**Published:** 2023-04-12

**Authors:** Joshua Au Yeung, Zeljko Kraljevic, Akish Luintel, Alfred Balston, Esther Idowu, Richard J. Dobson, James T. Teo

**Affiliations:** ^1^ Department of Neuroscience, Kings College Hospital, London, United Kingdom; ^2^ Guys & St Thomas Hospital, London, United Kingdom; ^3^ Department of Biostatistics, Kings College London, London, United Kingdom; ^4^ NIHR Biomedical Research Centre, South London and Maudsley NHS Foundation Trust and King's College London, London, United Kingdom

**Keywords:** large language models, chatbot, natural language processing (computer science), digital health, AI safety, transformer

## Abstract

As large language models (LLMs) expand and become more advanced, so do the natural language processing capabilities of conversational AI, or “chatbots”. OpenAI's recent release, ChatGPT, uses a transformer-based model to enable human-like text generation and question-answering on general domain knowledge, while a healthcare-specific Large Language Model (LLM) such as GatorTron has focused on the real-world healthcare domain knowledge. As LLMs advance to achieve near human-level performances on medical question and answering benchmarks, it is probable that Conversational AI will soon be developed for use in healthcare. In this article we discuss the potential and compare the performance of two different approaches to generative pretrained transformers—ChatGPT, the most widely used general conversational LLM, and Foresight, a GPT (generative pretrained transformer) based model focused on modelling patients and disorders. The comparison is conducted on the task of forecasting relevant diagnoses based on clinical vignettes. We also discuss important considerations and limitations of transformer-based chatbots for clinical use.

## Background

In 2022, a Cambrian explosion of natural language processing (NLP) models flooded the machine learning field, from OpenAI's GPT3 (1) to Google's PALM (2), Gopher (3) and Chinchilla (4). Currently, NLP chatbots in healthcare primarily use rules-based, tree-based or Bayesian algorithms [like Babylon Health's algorithm (5) and other proprietary approaches]. The latest generation of NLP models are almost all exclusively based on the transformer model. Transformers are a type of artificial intelligence architecture introduced by Google in 2017, that achieved state-of-the-art performance on a wide range of NLP tasks (6). Transformers adopt a novel mechanism called “self-attention”, differentially weighting the significance of each part of the input data (e.g., text). Transformer-based NLP models trained on vast amounts of text data result in large language models (LLM) that have advanced capabilities beyond extractive or summarisation tasks, but also natural language generation. These models have the potential to be used as conversational AI or chatbots in healthcare.

**Figure 1:**
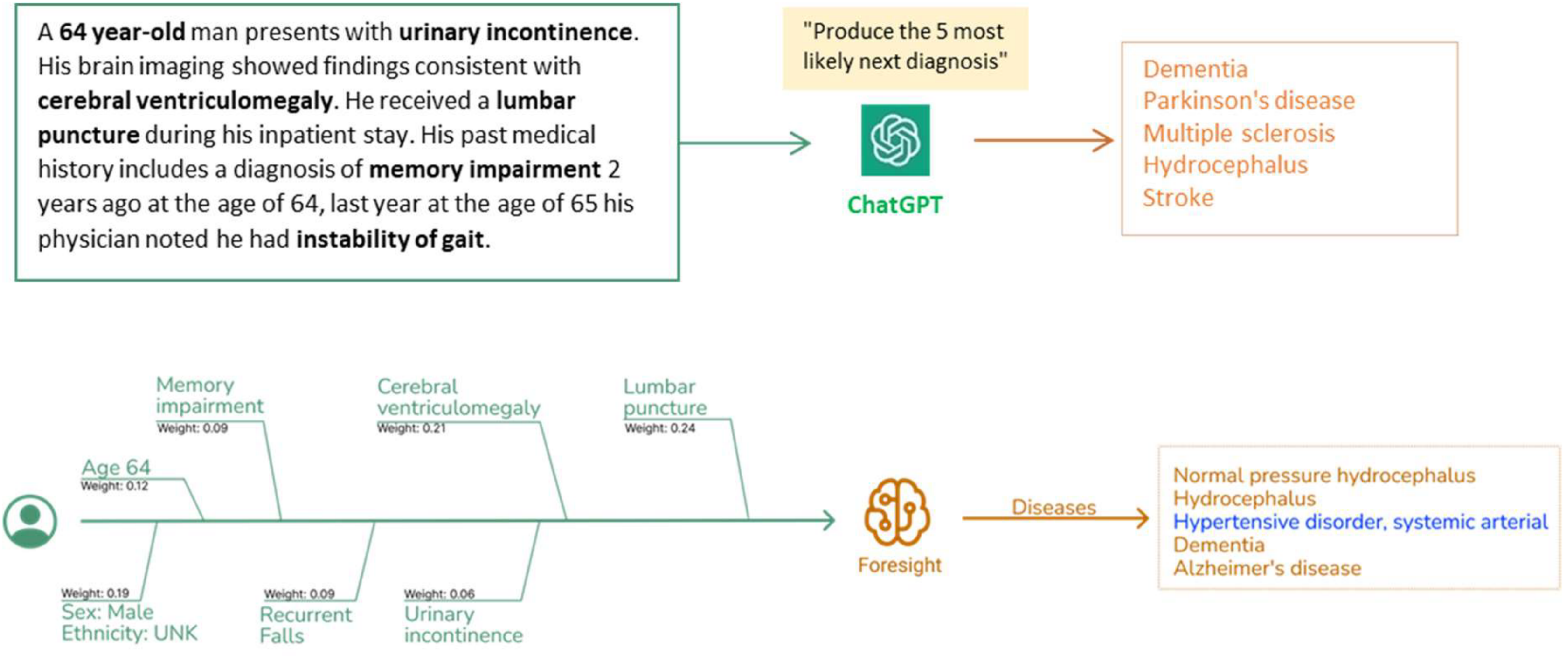
Clinical vignettes were inputted as prompts into transformer-based models: ChatGPT and foresight GPT model to generate outputs of the top 5 differential diagnoses.

As large language models (LLM) grow larger, their NLP capabilities become more advanced (3), leading to the development of emergent properties; the ability to perform tasks that it was not explicitly trained on (1). This is an advancement not seen in previous smaller language models and likely reflects the model's ability to learn and extract more knowledge from its training data. Transformer-based LLMs have demonstrated close to human-level performances in medical question and answering benchmarks and summarisation tasks (7–9), and with techniques like self-consistency (9), chain of thought prompting (10), and reinforcement learning from human feedback (11) the model performance can be further enhanced. Given their rapid rate of advancement, it is probable that LLM based conversational AI (chatbots) will soon be developed for healthcare use.

## Beyond the Turing test

While LLMs show promise in generating eloquent text outputs, patient safety and accuracy ranks higher in priority than human-like interactivity (ala Turing Test) in the healthcare domain. Also, the consideration should also apply for whether the tool is used by a clinician user (as clinical decision support) vs. the patient user (as an interactive medical chatbot).

LLMs like OpenAI's ChatGPT, have a broad knowledge representation through scouring the open internet, however potential limitations relate to them mirroring biases, associations and lack of accurate detail in the web-based training content (12). Alternatively, more curated approaches include training a LLM only on biomedical corpus datasets like Galactica (7) or PubMedGPT (8) to create a LLM with scientific domain-specific knowledge, but this captures biomedical publishing trends rather than trends of actual patients and diseases in healthcare. There are few LLMs that are trained and validated on real-world clinical data due to sensitivity of patient data and the significant computing power required to train these models. Methods to mitigate breaches of sensitive patient information include training a model on disease classification codes [e.g., BEHRT (13)] or on de-identified clinical notes [e.g., GatorTron (12)].

## Who is a large language model for?

Many biomedical LLM's have focused their performance against benchmark multiple-choice-question-(MCQ)-like tasks used in medical licensing examinations rather than for intended utility (14–16). These questions invariably are in medical jargon and answer academic scenarios when the actual needs of healthcare professionals are different: which is standardised information extraction from a specific (but voluminous) patient's record to support their human decision-making (17). The practical use of these models lies in their ability to support healthcare professionals in decision making from large unstructured patient records, unfortunately few models have been tested and validated on such tasks and on real-world hospital data.

An alternative approach we demonstrate is to train a LLM to map patient records onto a standardised ontology (SNOMED-CT) (18) and then to produce probabilistic forecasts from a specific record as a prompt. Primarily aimed at healthcare users, a demonstration web app is available at: https://foresight.sites.er.kcl.ac.uk/ (18).

To simulate a real-world scenario, it is not straightforward to evaluate performance since existing medical AI benchmark Q&A datasets do not actually reflect real-world clinical practice—a medical professional doesn't choose one correct diagnosis, but instead produces a list of ranked differential diagnoses which are all concurrently investigated for, treated for, and then progressively eliminated as more information becomes available. Relevancy and relative uncertainty are what clinicians do in day-to-day practice rather than what is most “correct”. There have been attempts to create more diverse benchmarking sets such as MultiMedQA, a benchmark spanning medical exam, medical research, and consumer medical questions, as well as incorporating a human evaluation framework (9), but still more work is needed to fully address the evaluation of biomedical models.

As such, we've crafted synthetic clinical histories (in the style of a vignette) and tasked the models to predict the 5 most likely diagnoses. The vignettes were provided as prompts to two generative pretrained transformer models– ChatGPT (OpenAI, Dec 2022 version release), currently the most widely used and publicly available LLM, and Foresight GPT (King's College London, version 1.0 KCH model), a model trained on real-world hospital data (Figure 1). Generative pre-trained transformers (GPT) are a type of transformer model that is used to predict the next token given an input sequence. 5 clinicians then scored the relevancy of each forecasted output, and also recorded whether any crucial diagnoses were missing. Relevancy was chosen over Accuracy since there were frequent disagreements on Ground Truth and which of the forecasted concepts was most “correct”.

**Figure 2:**
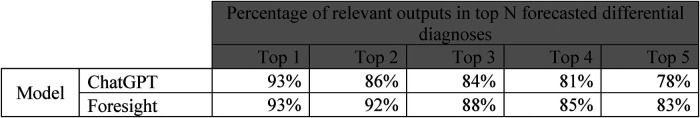
Table showing manual clinician evaluation of transformer-based model outputs on 35 imaginary patient vignettes. Columns represent number of relevant differential diagnoses in top N forecasted outputs.

Both models had high quantitative performance, with slightly superior performance in Foresight compared to ChatGPT for relevancy (93% vs. 93% relevancy in the top-1, 83% vs. 78% in the top 5 forecasted concepts) (Figure 2). However, clinicians reported that 21 out of 35 (60%) vignettes outputs from ChatGPT contained one or more crucial missing diagnoses (Supplementary C), which is unsurprising since ChatGPT is not domain-specific. Qualitatively, ChatGPT provides a substantially more eloquent free text generation but often with superficial high-level disease prediction categories instead of specific diseases (e.g., cardiac arrhythmia), while Foresight outputs more specific suggestions as diagnostic codes (e.g., right bundle branch block).

## Biases, hallucinations and falsehood mimicry

LLMs exhibit the same biases and associations of the web-based training text (19)—for example LLMs trained on Wikipedia and online news articles have been shown exhibit considerable levels of bias against particular country names, genders, and occupations (20). GPT-3 has been shown to exhibit gender-occupation associations, as well as negative sentiments with the Black race (1). Despite the moderation layer, in a scenario of analgesia choice for chest pain for a White patient compared with a Black patient, both ChatGPT and Foresight offered weaker analgesic treatment to the Black patient (Supplementary A). This could be due to clinician bias; racial-ethnic disparities in analgesic prescribing has been previously observed (21). Foresight also displayed an additional association where cocaine substance use was offered as a cause of chest pain in the White patient scenario (likely reflecting biases in the training population with cocaine-induced coronary vasospasm). Such sociodemographic differences in the source data as well as true genetic or ethnic risk is likely to show up in these deep learning models.

LLMs generate “hallucinations” whereby output is nonsensical or unfaithful to the provided input or “prompt” (22). Insufficient or masked information in the prompt (commonplace in the real-world healthcare) amplifies this issue yet ChatGPT produces high levels of confidence in its output. The confident natural language used in human-computer interaction by conversational AI may lead users to think of these agents as human-like (23). Anthropomorphising chatbots may inflate user' estimates of their knowledge and competency, this could lead to users blindly trusting chatbots output even if it contains unfaithful or factually incorrect information (19).

ChatGPT also takes the truth of prompts at face-value and is therefore susceptible to “Falsehood Mimicry” (Supplementary B); this is frequently demonstrated when a user inputs a factually incorrect prompt to ChatGPT, it will attempt to generate an output that fits the user's assumption instead of offering clarifying questions or a factual correction. Alternatively, Foresight produces a more transparent output with saliency maps and the level of uncertainty determined from relative probabilities of the differential diagnoses. Without an adequately skilled “prompter” or an “astute user”, a generative language AI may hallucinate misleading outputs with high certainty that can perpetuate harmful health beliefs, reinforce biases, or pose significant clinical risk.

## Future direction for generative language AI

Despite the promising advancement of LLMs and their sophistication in natural language processing and generation, our brief tests have highlighted the lack of readiness of transformer-based chatbots for use as a patient-facing clinical tool in its current form. LLMs have risks relating to the associations and biases of its training data, as well as the propensity to generate unfaithful or factually incorrect outputs. We believe the route to safe and responsible adoption of AI chatbots in healthcare will be through domain-specific training data scope (i.e., real world healthcare data and medical guidelines vs. biomedical training data), fine-tuning (e.g., RLHF) by expert clinicians mitigating risk through transparent representation of output relevancy vs. safety impact, and targeting a safer and more “skilled” end-user (the healthcare provider and not the patient).

## Data Availability

The original contributions presented in the study are included in the article/**Supplementary Material**, further inquiries can be directed to the corresponding author.
